# Distinct effects of apathy and dopamine on effort-based decision-making in Parkinson’s disease

**DOI:** 10.1093/brain/awy110

**Published:** 2018-04-23

**Authors:** Campbell Le Heron, Olivia Plant, Sanjay Manohar, Yuen-Siang Ang, Matthew Jackson, Graham Lennox, Michele T Hu, Masud Husain

**Affiliations:** 1 Nuffield Department of Clinical Neurosciences, University of Oxford, Oxford, UK; 2 Department of Experimental Psychology, University of Oxford, Oxford, UK; 3 Department of Neurology, John Radcliffe Hospital, Oxford University Hospitals Trust, Oxford, UK

**Keywords:** apathy, dopamine, decision-making, reward, Parkinson’s disease

## Abstract

Effort-based decision-making is a cognitive process crucial to normal motivated behaviour. Apathy is a common and disabling complication of Parkinson’s disease, but its aetiology remains unclear. Intriguingly, the neural substrates associated with apathy also subserve effort-based decision-making in animal models and humans. Furthermore, the dopaminergic system plays a core role in motivating effortful behaviour for reward, and its dysfunction has been proposed to play a crucial role in the aetiology of apathy in Parkinson’s disease. We hypothesized that disrupted effort-based decision-making underlies the syndrome of apathy in Parkinson’s disease, and that this disruption may be modulated by the dopaminergic system. An effort-based decision-making task was administered to 39 patients with Parkinson’s disease, with and without clinical apathy, ON and OFF their normal dopaminergic medications across two separate sessions, as well as 32 healthy age- and gender-matched controls. On a trial-by-trial basis, participants decided whether to accept or reject offers of monetary reward in return for exerting different levels of physical effort via handheld, individually calibrated dynamometers. Effort and reward were manipulated independently, such that offers spanned the full range of effort/reward combinations. Apathy was assessed using the Lille apathy rating scale. Motor effects of the dopamine manipulation were assessed using the Unified Parkinson’s Disease Rating Scale part three motor score. The primary outcome variable was choice (accept/decline offer) analysed using a hierarchical generalized linear mixed effects model, and the vigour of squeeze (Newtons exerted above required force). Both apathy and dopamine depletion were associated with reduced acceptance of offers. However, these effects were driven by dissociable patterns of responding. While apathy was characterized by increased rejection of predominantly low reward offers, dopamine increased responding to high effort, high reward offers, irrespective of underlying motivational state. Dopamine also exerted a main effect on motor vigour, increasing force production independently of reward offered, while apathy did not affect this measure. The findings demonstrate that disrupted effort-based decision-making underlies Parkinson’s disease apathy, but in a manner distinct to that caused by dopamine depletion. Apathy is associated with reduced incentivization by the rewarding outcomes of actions. In contrast, dopamine has a general effect in motivating behaviour for high effort, high reward options without altering the response pattern that characterizes the apathetic state. Thus, the motivational deficit observed in Parkinson’s disease appears not to be simply secondary to dopaminergic depletion of mesocorticolimbic pathways, suggesting non-dopaminergic therapeutic strategies for apathy may be important future targets.

## Teaser

The cognitive mechanisms underlying apathy in Parkinson’s disease are poorly understood. Le Heron *et al.* report that apathetic patients make decisions about effortful actions differently, and that dopamine does not correct this change. This finding sheds light on the causes of apathy, and suggests the importance of non-dopaminergic treatment strategies.

## Introduction

Deciding if an action, or sequence of actions, is ‘worth it’ is a cognitive process lying at the core of motivated goal-directed behaviour (Mogenson *et al.*, 1980; Balleine and O’Doherty, 2010; Salamone *et al.*, 2016). Often termed effort-based decision-making, the crucial evaluation is whether the rewarding outcomes of a potential behaviour outweigh its associated action costs (Kurniawan *et al.*, 2011). Although time delays and reward probability also determine the overall cost, there is good evidence that effort-related costs (whether physical or cognitive) are processed and integrated with reward information in distinct brain areas to drive choice (Rudebeck *et al.*, 2006; Prevost *et al.*, 2010; Rushworth *et al.*, 2012; Klein-Flugge *et al.*, 2016; Chong *et al.*, 2017; Hauser *et al.*, 2017).

In Parkinson’s disease, apathy or loss of motivation is a frequent and disabling complication (Aarsland *et al.*, 2009; Martinez-Fernandez *et al.*, 2016). Apathetic patients often report ‘I just can’t be bothered’ but a mechanistic understanding of this syndrome remains elusive*.* Although apathy has been conceptualized as a disorder of goal-directed behaviour for many years, a more robust understanding of its underlying neurocognitive mechanisms is lacking (Marin, 1991; Levy and Dubois, 2006; Starkstein and Leentjens, 2008). In Parkinson’s disease, apathy is associated in particular with reduced metabolism within ventral striatum, medial frontal cortex including dorsal anterior cingulate cortex and ventral midbrain (Lawrence *et al.*, 2011; Huang *et al.*, 2013; Robert *et al.*, 2014), reduced grey matter volume of ventral striatum (Carriere *et al.*, 2014), and reduced functional connectivity between these brain regions (Baggio *et al.*, 2015). Strikingly, these are the same brain areas demonstrated to underlie effort-based decision-making in healthy humans (Croxson *et al.*, 2009; Prevost *et al.*, 2010; Klein-Flugge *et al.*, 2016; Hauser *et al.*, 2017; Le Heron *et al.*, 2017). Furthermore, in experimental animal models, disruption of homologues of these regions induces a seemingly apathetic state, in which animals are no longer as willing to invest effort for reward (Walton *et al.*, 2002; Hauber and Sommer, 2009).

Aside from these anatomical considerations, there is some evidence that effort-based decision-making and Parkinson’s disease apathy might also share a common association with the neuromodulatory dopaminergic system. Dopamine projections from the ventral tegmental area particularly to ventral striatum and medial frontal cortex play a crucial role in effort-based decision-making, signalling the value of potential actions to drive appetitive/approach behaviours for reward (Kurniawan *et al.*, 2011; Salamone and Correa, 2012; Hamid *et al.*, 2016; Salamone *et al.*, 2016; Syed *et al.*, 2016). Disruption of this system with dopamine antagonists produces similar effects on effort-based decision-making as lesions to these regions, biasing animals away from high effort for high reward options (Salamone *et al.*, 2007). Reduced mesolimbic dopamine has also been hypothesized to contribute to apathy in Parkinson’s disease. These patients show reduced ventral striatum dopamine binding capacity, as well as blunted dopamine release following administration of methylphenidate, compared to Parkinson’s disease patients without apathy (Remy *et al.*, 2005; Thobois *et al.*, 2010; Santangelo *et al.*, 2015).

Such findings have motivated therapeutic trials of dopamine replacement for Parkinson’s disease apathy. However, results to date have been mixed and interpretation confounded by factors such as dopamine withdrawal following deep brain stimulation (Thobois *et al.*, 2013; Chung *et al.*, 2016). Thus, it remains unclear whether alterations in the dopaminergic system alone are responsible for the loss of motivation observed in Parkinson’s disease apathy. Indeed, recent findings point to a potential role for non-dopaminergic modulation of apathy in Parkinson’s disease, involving the serotonergic and cholinergic systems (Devos *et al.*, 2014; Maillet *et al.*, 2016). Effort-based decision-making in healthy people has also been shown to be affected by a serotonin reuptake inhibitor (Meyniel *et al.*, 2016), while an important role for adenosine A_2A_ receptors in this process has been demonstrated in rodents (Nunes *et al.*, 2013). These findings raise the possibility that non-dopaminergic neurotransmitter systems normally play an important role in guiding such behaviour (Salamone *et al.*, 2016).

Here, we aim to investigate the relationship between effort-based decision-making and Parkinson’s disease apathy, as well as the impact of dopamine therapy, within the same experimental framework. We hypothesize that disruption of effort-based decision-making—specifically the mechanism by which individuals weigh up rewards and costs associated with a potential action or sequence of actions—provides a plausible explanation for the reduced goal-directed behaviour that defines apathy in Parkinson’s disease. Furthermore, by examining patients ON and OFF their dopaminergic medication, we can investigate whether this disruption in effort-based decision-making might indeed be mediated by alterations in dopaminergic tone.

The findings of some recent studies provide further motivation for this approach. First, it has been shown that apathy in Parkinson’s disease is associated with reduced autonomic (Muhammed *et al.*, 2016) and electrophysiological (Martinez-Horta *et al.*, 2014) responses to reward, consistent with a failure of incentivization by reward. However, those studies did not examine decision-making based on weighing up the effort required to obtain a particular reward. Second, it has been reported that Parkinson’s disease patients when tested ON dopaminergic medication are prepared to exert more effort for a given reward than when OFF medication (after an overnight withdrawal of these drugs) (Chong *et al.*, 2015; Le Bouc *et al.*, 2016). However, patients in those studies had normal levels of motivation and it is unclear whether these findings would translate into a group who are clinically apathetic, or whether in fact the motivational effects of dopamine occur on a separate axis to the deficits that underlie apathy. This is important because apathetic behaviour might result either from hypersensitivity to the effort costs of actions, insensitivity to the rewarding outcomes of actions or a combination of both. Moreover, the effects of dopamine might be different to the effects of apathy.

We administered an effort-based decision-making task to Parkinson’s disease patients with and without clinical apathy, ON and OFF their normal dopaminergic medications, to assess the independent effects of these factors on effort-based decision-making, as well as the interaction between the two. Furthermore, our experimental design allowed us to dissociate the distinct effects of effort and reward on choice behaviour within a biologically relevant decision space. This potentially provides a deeper mechanistic understanding of any differences we identify, but also enables translation of findings back to everyday behaviour. Specifically, we investigated whether changes in effort-based decision-making associated with apathy, and being OFF dopamine, manifest globally across decision space (e.g. as a generalized reduction in engagement with the task) or whether these factors have more specific, dissociable effects. This approach allowed us to examine whether dopamine and apathy have the same or distinctly different effects on effort-based decision-making.

## Materials and methods

### Ethics

The study was approved by the local ethics committee and written consent was obtained from all subjects in accordance with the Declaration of Helsinki.

### Participants

Forty-one patients with a clinical diagnosis of idiopathic Parkinson’s disease (confirmed by two neurologists: C.L.H. and either M.J., G.L., M.T.H. or M.H.) were recruited from local movement disorders clinics in the Oxfordshire area, UK. Inclusion criteria included an absence of Parkinson’s disease dementia or other major neurological or psychiatric conditions, although the presence of depression was not a specific exclusion factor. We specifically aimed to recruit an even sample of apathetic and non-apathetic patients. Two patients were subsequently excluded—one because of a failure to understand the task, and one who did not attend the second session, leaving 39 patients. Thirty-two healthy age- and gender-matched controls were recruited via a local database. All patients were tested in two morning sessions, one ON their normal dopaminergic medications and the other having withheld these since the night before (OFF), counterbalanced across apathy status.

### Disease, cognitive and questionnaire measures

Apathy was assessed by standardized clinical interview with the patient, using the Lille apathy rating scale (LARS: range −36 to 36), which has previously been validated in Parkinson’s disease (Sockeel *et al.*, 2006). Patients were classified as apathetic if their LARS score was >−22 (a cut-off corresponding to at least mild-moderate apathy levels). The LARS can also be divided into four subscales (termed intellectual curiosity, emotion, action initiation and self-awareness), each ranging from −4 to 4. Severity of Parkinson’s disease was assessed using the Unified Parkinson’s Disease Rating Scale (UPDRS) total score (Goetz *et al.*, 2008) and Hoehn and Yahr stage. The UPDRS-III (motor score) was repeated in the ON and OFF states. As a baseline cognitive screen, all subjects were administered the Addenbrooke’s cognitive examination version III (ACE-III) (Hsieh *et al.*, 2013), and a digit span task to assess working memory (Groth-Marnat, 2000), in the ON state. Depressive symptoms were assessed using the Beck Depression Inventory-II (BDI-II) (Beck *et al.*, 1996). A separate dysphoria-only subscale of the BDI-II was also calculated, given the potential for overlap between apathy and anhedonia components of depression (Kirsch-Darrow *et al.*, 2011; Treadway and Zald, 2011). Quality of life (QOL) was assessed using a Cantril ladder (Cantril, 1965). Demographics are displayed in Table 1.

**Table 1:** Demographic, questionnaire and baseline cognitive measures

Measure	Healthy elderly controls	Parkinson’s disease	Control versus Parkinson’s disease *P*-value	Parkinson’s disease, no apathy (LARS ≤−22)	Parkinson’s disease, apathy (LARS >−22)	No apathy versus apathy *P*-value
*n*	32	39	n/a	18	21	n/a
Age	68.9 (±6.9)	67.8 (±7.6)	0.55	68.2 (±6.5)	67.5 (±8.5)	0.78
Gender (F/M)	13/19	12/27	0.39^a^	8/10	4/17	0.16^a^
Apathy (LARS)	−28.3 (±3.1)	−20.9 (±6.8)	**<0.0001**	−27 (±3.8)	−15.7 (±3.8)	**<0.0001**
Hoehn and Yahr stage	n/a	2.2 (±0.51)	n/a	2.1 (±0.6)	2.2 (±0.4)	0.44
UPDRS total	n/a	82.3 (26.6)	n/a	73.8 (±25.7)	89.9 (±25.7)	0.08
UPDRS motor score ON	n/a	28.4 (±11.4)	n/a	27.1 (±13.2)	29.5 (±9.9)	0.5
UPDRS motor score OFF	n/a	35.9 (±10.8)	n/a	32.7 (±11.0)	38.7 (±10.2)	0.09
Change in motor score	n/a	7.5 (±8.3)	n/a	5.7 (±8.3)	9.1 (±8.1)	0.2
Levodopa equivalent dose (mg/24 h)	n/a	633 (±356)	n/a	565 (±353)	691 (±357)	0.27
Hours since last dose, OFF	n/a	18 (±3)	n/a	19 (±3)	17 (±3)	**0.04**
Hours since last dose, ON	n/a	2.3 (±1.5)	n/a	2.7 (±1.6)	2 (±1.4)	0.19
Depression (BDI-II)	3.8 (±3.7)	14.3 (±7.7)	**<0.0001**	11 (±7.0)	17.1 (±7.4)	**0.01**
Dysphoria subscale	1.1 (±1.5)	4.9 (±4.0)	**<0.0001**	4.4 (±4.2)	5.3 (±3.8)	0.53
Global Cognition (ACE)	95.6 (±3.8)	91.1 (±7.9)	**0.005**	93.5 (±5.0)	89.4 (±9.4)	0.08
Quality of life	8.0 (±1.2)	6.7 (±1.8)	**0.001**	7.6 (±1.5)	6.0 (±1.8)	**0.01**
Digit span	20.6 (±4.4)	17.4 (±3.6)	**0.002**	16.7 (±3.1)	17.9 (±3.9)	0.35

ACE = Addenbrooke’s cognitive examination III; BDI-II = Beck’s depression inventory II; n/a = not applicable; Quality of life = Cantril ladder (range 0–10).

^a^Chi-squared test; all values are mean (± standard deviation). Statistically significant comparisons are shown in bold.

### Experimental design

Participants were seated in front of a desktop computer running Psychtoolbox (psychtoolbox.org) implemented within MATLAB (MathWorks, USA). They registered their responses using one of two handheld dynamometers (SS25LA, BIOPAC Systems), which were calibrated to each individual. Each participant’s maximal voluntary contraction (MVC) was calculated separately for each hand and session, as the greatest force exerted over three maximal contractions. All subsequent responses were thus normalized to each individual.

**Figure 1:**
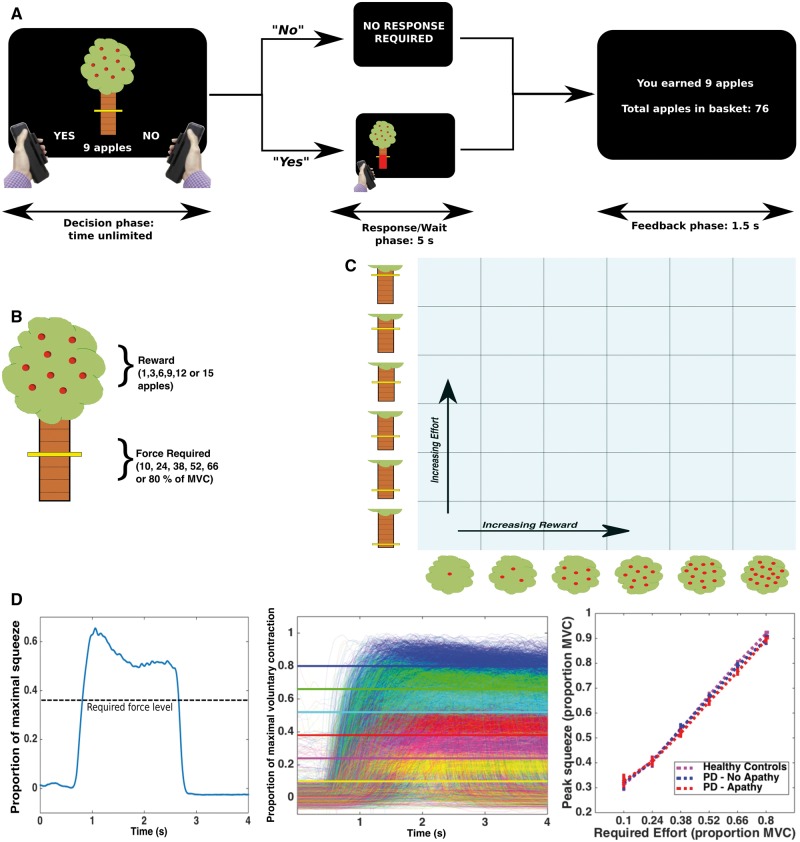
**Effort-based decision-making task.** On a trial-by-trial basis, participants were presented with offers of a certain amount of reward (apples on an apple tree, with each apple worth 1p) in return for a level of physical effort [ranging between 10% and 80% of a subjects’ previously determined maximal voluntary contraction (MVC), held for 1 s] (**A** and **B**). They were instructed to weigh up each offer, deciding ‘whether it was worth it’. If they accepted an offer (by squeezing the left-hand grip) the tree moved to the left or right of the screen, indicating which hand they had to perform the force squeeze with. They had a 5-s window within which to achieve the required force level. If they rejected the offer (by squeezing the right-hand grip) they waited the same 5-s period. After a feedback phase they then moved onto the next trial. After a practice session, participants worked through 180 trials, which evenly sampled the 6 × 6 ‘decision space’ over five blocks (**C**). Example force trace from a single trial (**D**, *left).* Participants parametrically modulated force output to task requirements. Superimposed force traces for all patients, ON and OFF their dopaminergic medications. Each required force level is a different colour, and the solid lines show the minimum required level (**D**, *middle*). Both patients and healthy controls modulated squeeze force appropriately (**D**, *right*). PD = Parkinson’s disease.

On a trial-by-trial basis, participants were given sequential offers of reward in return for exerting effort (Fig. 1A). Each offer was presented on the screen as a cartoon apple tree. Reward on offer for the current trial was indicated by the number of apples on the tree (1, 3, 6, 9, 12 or 15, with each apple worth 1p), while effort required to obtain this reward was indicated by the height of a yellow bar on the tree trunk (levels corresponded to 10, 24, 38, 52, 66 and 80% of the participant’s MVC; Fig. 1B). The six reward and effort levels were systematically combined and the 36 conditions sampled evenly in a pseudo-randomized order across five blocks, for a total of 180 trials (Fig. 1C). This meant all participants received the same offers presented in the same order.

Participants were instructed to weigh up the effort costs against the reward offered for each trial, and decide ‘whether it is worth it’*.* If they accepted an offer (by exerting a small squeeze on the left hand-grip) then they had to squeeze to the required force and hold this for >1 s (within a 5-s response window), before being ‘rewarded’ with the apples. During the squeeze, online force feedback was shown as a red bar, indicating current force relative to the target line. Conversely, if participants rejected the offer (via a small squeeze on the right hand-grip) they waited an equivalent time (to avoid possible effects of temporal discounting) before moving onto the next offer. Therefore, on each trial, participants decided whether the value of an offer was worth engaging with, compared to doing nothing for the equivalent time (Fig. 1A).

Before starting the experiment, participants practised each force level with each hand to familiarize themselves with the effort required, and completed a practice block in which they made decisions on the full range of options in the experiment. They were informed that each apple collected was worth 1p, and that, in addition to a flat rate to cover their expenses and time, they could earn additional money based on apples collected. Instructions were presented in ‘neutral’ language (i.e. participants were not encouraged or discouraged to accept offers), rather they were asked to consider ‘for each offer, of a certain number of apples in return for exerting a certain level of physical effort, is it worth it, is it worth squeezing that hard for that number of apples?’ In reality, patients were paid a fixed sum of £10 for the experiment, as part of a larger payment of £30–40 to cover expenses; however, no specific performance feedback was given.

We included a number of features to reduce potential effects of fatigue on choice. Both hands were used for the experiment. The required response side was randomized, and signalled on each trial after an offer was accepted, by the apple tree moving from the centre position to the left (left hand) or right (right hand) side of the screen, selected pseudo-randomly on each trial. Additionally, 25% of accepted offers did not require a squeeze (although subjects were instructed to make their responses assuming they would have to exert effort if accepted); in these cases (which were pseudo-randomly distributed throughout the experiment) subjects waited the equivalent time as if they had rejected the offer. Finally, subjects were allowed to rest between each block of 36 trials until they felt ready to continue (generally 30–120 s).

In addition to choice (accept/reject), decision latency and force metrics (sampled at 500 Hz) were recorded (Fig. 1D) for each trial. We used the peak force exerted on each trial (in Newtons) as a measure of motor vigour (Le Bouc *et al.*, 2016), normalizing this value across effort levels by subtracting the minimum required force level for that particular trial (Fig. 4A). Following the experiment, participants rated how physically challenging they found each of the effort levels on a 21-point visual rating scale.

### Statistical analysis

Data were analysed within MATLAB (MathWorks, USA) using custom scripts. Trials where decision time was <0.4 s were discarded as accidental squeezes. Because of the hierarchical nature of our study, we used generalized linear mixed effects with a logistic link function to model accept/reject responses (*fitglme* within MATLAB) for the primary analysis. We included a random effect of subject, and fixed effects of reward, effort, apathy status and dopamine state. We also included a main effect of session (first or second) as this parameter significantly improved model fit (change in Akaike Information Criterion units = 14). Notably, a session × drug interaction term (ON-OFF versus OFF-ON order) was not significant and worsened model fit (*P* = 0.48, increase in Akaike Information Criterion units = 1.5) and was not included in the model. The inclusion or exclusion of the session and session × drug terms did not affect any other results.

Our main approach was to fit the full model (including all interactions between reward, effort, apathy and dopamine). We compared this model to all other possible combinations, using the Akaike Information Criterion. No model improved fit by >2 Akaike Information Criterion units (a standard cut-off level, Burnham and Anderson, 2004), and we therefore report the full model results (see Supplementary material, Supplementary Table 1 and Supplementary Fig. 1 for further details).

Here we report the *t*-statistic, *F*-statistic and *P*-value associated with each parameter estimate. All statistical tests were two-tailed, using an alpha-level of *P < *0.05.

Additionally, to answer the initial question of whether there was an overall difference in offer acceptance associated with either apathy or dopamine depletion, we used the proportion of offers accepted as a raw measure of task performance. The baseline effect of apathy on choice was examined with subjects in their normal ON state, using a one-way ANOVA for the three groups (healthy controls, Parkinson’s disease non-apathetic cases; and Parkinson’s disease apathetic cases). The main effect of dopamine on this metric was calculated using a paired *t*-test of change in proportion acceptance ON versus OFF. Change in motor vigour was analysed using a repeated measures ANOVA with dopamine as a within subject variable, apathy between subject and force (Newtons) the dependent variable. Tests for differences in demographic factors between groups were performed using standard unpaired *t*-tests, or, when it was of interest to include healthy controls, Parkinson’s disease with apathy and Parkinson’s disease without apathy together, a one-way ANOVA.

## Results

### Clinical apathy and dopamine depletion reduce offer acceptance

**Figure 2:**
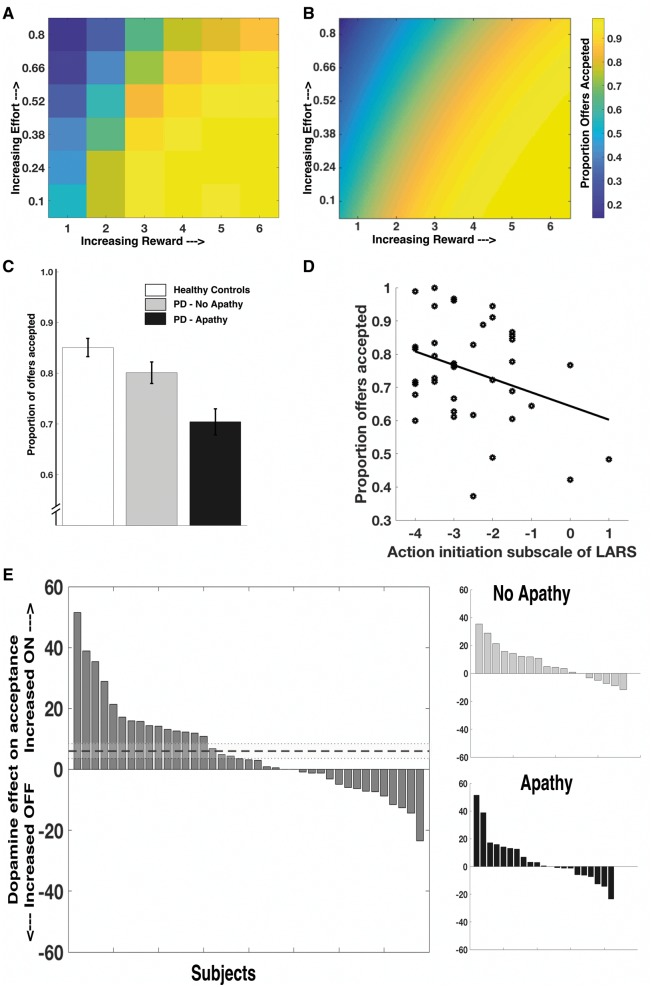
**Raw acceptance results as apathy status, and dopamine state, varied.** Mean acceptance rates for all patients, ON and OFF their normal medications. As reward on offer increased, and effort required decreased, the probability of a participant accepting an offer increased, raw data (**A**) and modelled data (**B**). Apathetic Parkinson’s disease patients accepted significantly fewer offers than healthy controls or non-apathetic Parkinson’s disease patients (**C**, ON state shown, error bars are ± SEM). Apathy level (action initiation subscale of LARS) strongly correlated with acceptance rate (**D**, r = 0.37, *P = *0.019; ON state shown). Dopamine depletion (OFF state) was associated with significantly reduced acceptance rates, irrespective of apathy status (**E**, dotted line is mean effect ± SEM). PD = Parkinson’s disease.

We first assessed the raw effects of clinical apathy on choice by comparing group performance in the ON state. There was a significant main effect of group (healthy controls, Parkinson’s disease without apathy and Parkinson’s disease with apathy) on number of offers accepted [*F*(2,68) = 8.12, *P = *0.001, Fig. 2C]. Apathetic Parkinson’s disease patients accepted significantly fewer offers than both healthy controls [mean difference in proportion of offers accepted 0.15, *t*(51) = 4.01, *P < *0.001] and non-apathetic Parkinson’s disease patients [mean difference 0.10, *t*(37) = 2.33, *P = *0.029] across the experiment. There was no significant difference between non-apathetic Parkinson’s disease patients and healthy controls [mean difference 0.049, *t*(48) = 1.3, *P = *0.20]. Task performance correlated most closely with the action initiation (AI) subscale of the LARS (r = 0.37, *n* = 39, *P = *0.019, Fig. 2D and Supplementary Fig. 3). Thus the more apathetic an individual was with respect to action initiation, the less likely they were to accept an offer on the task. This association could not be explained by motor deficit, with no significant relationship between acceptance rate and UPDRS motor score (R = 0.14, *n = *39, *P = *0.38, Supplementary Fig. 4).

Dopamine depletion in these Parkinson’s disease patients, as indexed by performance when OFF dopaminergic drugs, also reduced offer acceptance. Patients accepted significantly more offers when ON compared to OFF their usual dopaminergic medications [mean increase ON dopamine 6% (SEM 2.4%), *t*(38) = 2.45, *P = *0.019; Fig. 2E]. Interestingly, although overall dopamine depletion reduced offers accepted, this effect was quite heterogeneous in the population, with some individuals (from both sides of the apathy spectrum) actually accepting more offers in the OFF state (Fig. 2E). There was no correlation between the AI (action initiation) subscale of the LARS and effect of dopamine on acceptance rate (R = 0.06, *n* = 39, *P = *0.71, Supplementary Fig. 4). These findings therefore show that both apathy and being OFF dopaminergic medication (regardless of apathy status) reduce the willingness to invest effort for reward.

### Distinct interactions drive reduced responding in apathy and dopamine depletion

To determine the driving factors behind the reduced responding associated with both apathy and dopamine depletion, we used a generalized linear mixed effects model approach (see ‘Materials and methods’ section and Supplementary material). There were two significant, independent three-way interactions: (i) Apathy × Reward × Effort [t = 3.02; *F*(1,39) = 9.15; *P = *0.004]; and (ii) Dopamine × Reward × Effort [t = −3.14; *F*(1,39) = 9.84; *P* = 0.003].

**Figure 3:**
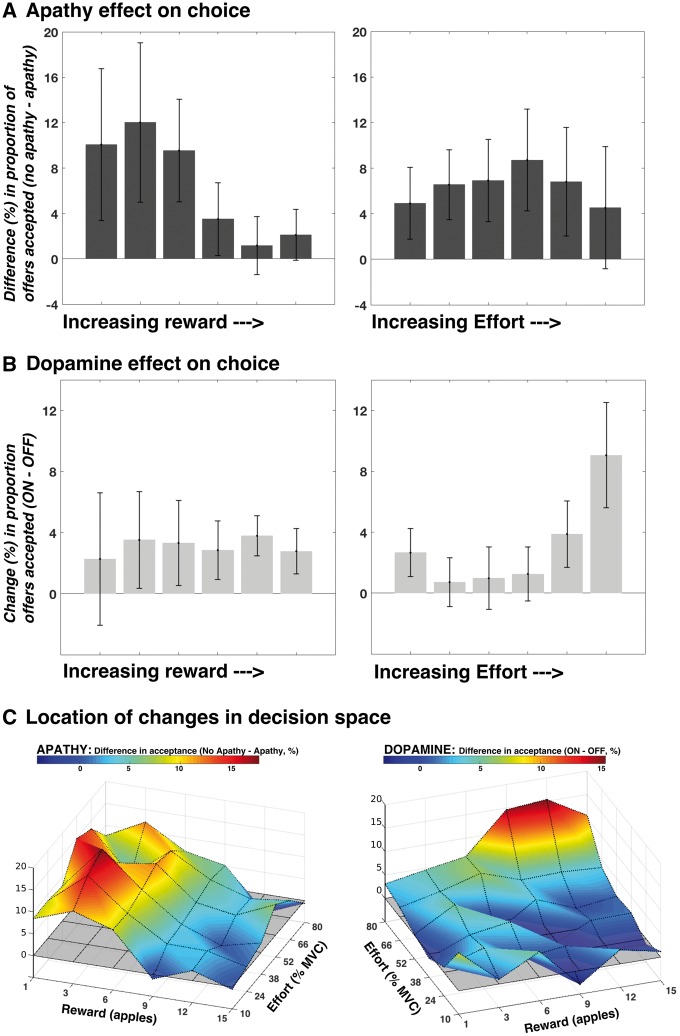
**Distinct effects of apathy and dopamine depletion on response pattern.** Change in acceptance in apathetic compared to non-apathetic patients, as both reward and effort increase. The panels show difference in responding (% accepted in no apathy group − % accepted in apathy group) at each reward level, collapsed across effort (**A**, *left*) and effort level, collapsed across reward (**A**, *right*). The reduced acceptance rate seen in apathetic patients was driven by reduced responding to low reward offers, particularly when effort costs were lower (Apathy × Reward × Effort interaction *P = *0.004). A different behavioural pattern characterized the effects of dopamine depletion (OFF state) on choice. Panels show the mean change in acceptance (ON minus OFF) across patients, as reward (**B**, *left*) and effort (**B**, right) increases. The difference between ON and OFF states was driven by increased responding to predominantly high effort, higher reward offers (Dopamine × Reward × Effort interaction *P = *0.003). These differences in acceptance rates associated with apathy (**C**, *left*) and dopamine depletion (**C**, *right*) manifest within distinct regions of the 6 × 6 decision space grid. The grey shaded plane (*z*-axis = 0) represents no difference in acceptance. All error bars are ± SEM. PD = Parkinson’s disease.

These indicate that the effects of apathy and dopamine on choice were not constant throughout decision space. Instead, apathy reduced responses for low reward offers, particularly when effort costs were lower, while dopamine depletion reduced responses for high effort, high reward offers (Fig. 3A and B). Figure 3C illustrates the distinct regions of decision space affected by the two variables. In addition to these effects, there were significant main effects of reward [t = 43; *F*(1,39) = 1849; *P < *0.0001], effort [t = −26.4; *F*(1,39) = 701; *P < *0.0001], dopamine [t = −3.38; *F*(1,39) = 11.4, *P < *0.0016], and session [t = 4.79, *F*(1,39) = 22.95, *P < *0.0001], although not apathy [t = −1.49; *F*(1,39) = 2.23, *P = *0.14], as well as a significant Dopamine × Effort interaction [t = −2.87; *F*(1,39) = 8.24, *P = *0.0066], Apathy × Effort interaction [t = 2.57; *F*(1,39) = 6.58, *P = *0.014] and Reward × Effort interaction [t = −7.89; *F*(1,39) = 62.3, *P < *0.0001]. There were no significant two-way interactions between reward and apathy (*P = *0.63), reward and dopamine (*P = *0.12), or apathy and dopamine (*P = *0.55), nor were the Apathy × Dopamine ×Effort, Apathy × Dopamine × Reward interactions, or the full four-way interaction significant (*P = *0.09, *P = *0.75 and *P = *0.27, respectively) (see Supplementary Fig. 1 for graphical illustration of these factors, and Supplementary Fig. 2 for the choice data split by reward and effort level).

### Dopamine depletion, but not apathy, reduces raw motor response for reward

**Figure 4:**
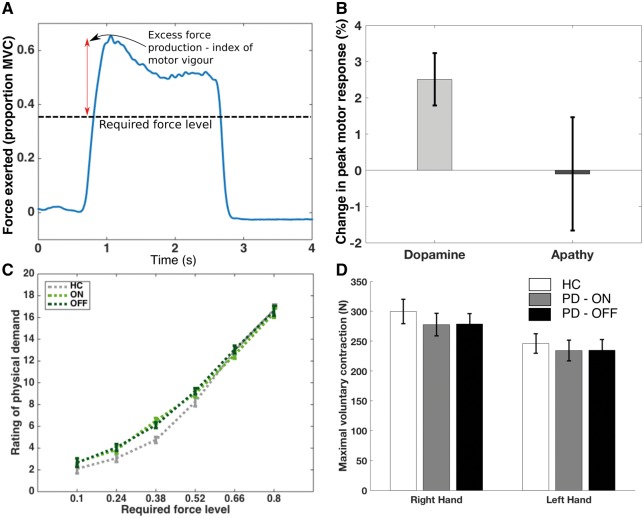
**Dopamine depletion, but not apathy, reduces motor vigour following decision to engage.** Motor vigour was indexed by excess force generated above the required effort level, i.e. how much more than was required that a participant squeezed (**A**). Dopamine (ON state) significantly increased response vigour (indexed by OFF state), while the presence of apathy was not associated with a significant change in the vigour of response (**B**). Despite greater force production at each effort level in the ON state, there was no change in perceived physical exertion between ON and OFF state, nor between patients and controls (**C**). Maximal voluntary contraction did not differ between ON and OFF states (**D**). PD = Parkinson’s disease.

Dopamine increased the peak squeeze force each patient exerted to obtain rewards, even though this increased output above the required force level was not coupled to any increase in reward [mean increase in peak force = 2.5% of subject’s MVC, SEM 0.72%, *t*(38) = 3.47, *P* = 0.0013; Fig. 4A and B]. This main effect of dopamine [*F*(1,31) = 9.25, *P = *0.005], was present for both high and low effort levels, with no interaction between effort and dopamine [*F*(5,155) = 1.4, *P = *0.24]. Importantly, this effect of dopamine was not modulated by apathy status [Apathy × Dopamine effect *F*(1,31) = 0.68, *P = *0.42], nor was there a main effect of apathy on force exerted at each effort level [*F*(1,31) = 0.25, *P = *0.62; Fig. 4B]. Thus, once apathetic patients had accepted an option, their subsequent peak motor response was no different to non-apathetic patients.

The subjective rating of the physical demand associated with each effort level did not differ between ON and OFF dopaminergic states, or between patients and controls [one-way ANOVA for group effect: *F*(2,95) = 0.6, *P = *0.56; Fig. 4C]. Additionally, apathetic subjects did not perceive effort as more demanding (Supplementary Fig. 5). Of note there was no significant change in MVC between ON and OFF states: mean for right hand [ON versus OFF = 277.9 N versus 278.8 N; *t*(38) = −0.12, *P = *0.9]; left hand [234.1 N versus 234.8 N; *t*(38) = −0.07, *P = *0.94; *P*-values on paired *t*-tests; Fig. 4D].

Participants in both control and Parkinson’s disease groups parametrically adjusted their force production to trial requirements (Fig. 1D). Of note, participants achieved the force requirements on the offers they accepted >95% of the time, and this did not differ between apathetic and non-apathetic patients [*t*(37) = −1.19, *P = *0.24] or the ON and OFF states [*t*(38) = −0.39, *P = *0.7; Supplementary Fig. 6]. Their choices did not systematically change across the experiment, with no effect of block number on choice in the ON state [*F*(4,179) = 0.03, *P = *0.99], OFF state [*F*(4,179) = 0.2, *P = *0.94], or for apathetic [*F*(4,179) = 0.11, *P = *0.99] and non-apathetic groups [*F*(4,179) = 0.04, *P = *0.98; Supplementary Fig. 7]. There was also no significant change in motor vigour between the beginning and end of the experiment (mean difference between first and fifth blocks = 0.008, *P = *0.21). Although vigour varied within the experiment, increasing during the first half of the experiment before returning towards baseline by the end, there was no Drug × Block interaction [*F*(3.1,118) = 0.98, *P = *0.41; Supplementary Fig. 8].

Decision time (time to accept or reject an offer) was strongly related to offer value (reward discounted by effort) for both apathetic and non-apathetic patients, and in ON and OFF states (Supplementary Fig. 9), suggesting all groups attended appropriately to the task. Apathetic patients took on average 1.67 s to make each decision, compared to 1.49 s for non-apathetic patients, although this difference was not statistically significant (*P = *0.35). Patients in the OFF state trended towards faster decisions than when in the ON state [mean(ON) = 1.67 s, mean(OFF) = 1.48 s, *F*(1,37) = 4.04, *P = *0.052; Supplementary Fig. 10]. There was no relationship between decision time and UPDRS motor score (ON): R = 0.17, *n = *39, *P = *0.30. There was also no relationship between change in UPDRS motor score and change in acceptance rate, ON and OFF dopamine (R = 0.15, *n = *39, *P = *0.35).

Willingness to accept offers was not affected by the presence of dysphoria (assessed using the dysphoria subscale of the BDI-II)—proportion of offers accepted in low dysphoria group* = *0.75 (±0.028); high dysphoria group* = *0.76 (±0.04), *t*(34) = 0.29, *P = *0.77 (Supplementary Fig. 11). Having Parkinson’s disease was associated with a lower quality of life compared to healthy controls; however, this change was entirely driven by a reduced quality of life in the apathetic group [overall group effect: *F*(2,65) = 11.4, *P < *0.001; Parkinson’s disease with apathy versus Parkinson’s disease no apathy: mean difference = 1.5, *P = *0.008, Parkinson’s disease with apathy versus healthy controls: mean difference = 2.0, *P < *0.001; Parkinson’s disease no apathy versus healthy controls: mean difference = 0.5, *P = *0.58].

## Discussion

This study demonstrates that altered effort-based decision-making is associated with the clinical phenotype of apathy in patients with Parkinson’s disease. The effect is not a generalized reduction in willingness to engage in effort, but rather is predominantly driven by reduced responding to low reward outcomes, suggesting apathy is associated with reduced incentivization by reward, rather than increased sensitivity to action costs. This finding provides a plausible mechanism for the reduced goal directed behaviour that characterizes the apathetic state. In contrast, the results demonstrate that although dopamine depletion also impacts upon effort-based decision-making, it does so in a distinctly different manner to apathy, affecting individuals’ willingness to accept predominantly high effort, high reward offers.

Whilst apathy has long been conceptualized as a disorder of goal-directed behaviour (Marin, 1991; Levy and Dubois, 2006), empirical evidence based on neurobiological accounts of this is sparse (but see Le Bouc *et al.*, 2016). The similarity of the apathy phenotype, across a diverse range of underlying pathologies, suggests a common neurocognitive system may be implicated in its aetiology. The brain network underlying effort-based decision-making is one such candidate system intrinsic to normal motivated behaviour, and indeed a recent neurocognitive framework of apathy emphasizes three core components of it that could potentially underlie the apathetic phenotype (Le Heron *et al.*, 2017). They include: (i) deciding whether to act; (ii) persisting with an action; and (iii) learning about the outcome of an action, and are considered to be instantiated within brain regions implicated in Parkinson’s disease apathy (Carriere *et al.*, 2014; Robert *et al.*, 2014; Baggio *et al.*, 2015), particularly ventral striatum and dorsal anterior cingulate cortex (Hauber and Sommer, 2009; Kolling *et al.*, 2016; Salamone *et al.*, 2016). Here we provide empirical evidence implicating the first of these component processes—deciding whether to act—in Parkinson’s disease apathy.

Importantly, this reduced responding is not global, but rather is limited to a subset of offers—those in which rewarding outcomes of actions are low (particularly when costs are low as well). Put another way, apathetic patients were prepared to exert effort—even high levels of effort—to the same degree as non-apathetic patients, if the rewarding outcome was high enough. In fact, apathetic patients showed reduced, rather than increased, effort sensitivity, despite modulating their force output in the same way as non-apathetic patients. These results suggest Parkinson’s disease apathy is associated with reduced incentivization by the rewarding outcomes of actions, rather than an increased sensitivity to effort costs *per se*. A growing body of evidence supports this observation. Parkinson’s disease apathy is associated with reduced autonomic (Schmidt *et al.*, 2008; Muhammed *et al.*, 2016) and neural (Lawrence *et al.*, 2011) responses to reward. Parkinson’s disease patients with apathy also show reduced electrophysiological responses (feedback related negativity) to rewarding and aversive outcomes, suggesting an impairment in underlying incentive processing (Martinez-Horta *et al.*, 2014). These reward-related signals are likely crucial for motivating behaviour towards a goal, overcoming the costs of action as an individual decides whether or not to act (Holroyd and Yeung, 2012; Rushworth *et al.*, 2012; Salamone *et al.*, 2016). More generally, this conceptualization suggests that the core feature of clinical apathy—reduced goal-directed behaviours—may manifest because patients no longer derive sufficient ‘drive’ from the outcomes of options in their everyday environment.

Effort-based decision-making was also affected by dopamine depletion. Specifically, we found that the reduced responding observed in the OFF state was driven by greater rejection of offers with higher associated effort costs and rewards. This finding is consistent with recent animal research that suggests a crucial role for mesolimbic dopamine in overcoming the costs of work, by signalling the value of actions towards a goal (Howe *et al.*, 2013; Hamid *et al.*, 2016; Syed *et al.*, 2016). More generally, it demonstrates the inextricable linkage that exists between the dopaminergic system and (motivated) goal-directed actions towards incentives. The result is concordant with previous work, which has demonstrated that manipulation of the dopaminergic system reduces effortful behaviour for rewards in animals (Salamone and Correa, 2012) and humans (Chong *et al.*, 2015; Le Bouc *et al.*, 2016), but extends it in two important directions.

First, we demonstrate that dopamine’s effect on effort-based decision-making was present across underlying motivational states. Both apathetic and non-apathetic Parkinson’s disease patients showed increased acceptance of high effort, high reward offers when ON compared to OFF their usual dopaminergic medications. Second, we demonstrate that dopamine depletion and apathy have dissociable effects on effort-based decision-making. The common occurrence of apathy in conditions such as Parkinson’s disease—in which a hypo-dopaminergic state is a defining feature (Aarsland *et al.*, 2009; Kalia and Lang, 2015)—as well as neuroimaging work in patients with Parkinson’s disease who have developed apathy in the context of deep brain stimulation (Thobois *et al.*, 2010) and *de novo* (Santangelo *et al.*, 2015) has led to the hypothesis that apathy in Parkinson’s disease occurs as the result of a dopaminergic denervation within the mesolimbic system (Thobois *et al.*, 2010). This has motivated therapeutic trials to treat apathy in Parkinson’s disease with further dopamine replacement. However, to date results have been mixed. A single randomized controlled trial suggested an improvement in apathy questionnaire measures with administration of the D2/3 receptor agonist piribedil, albeit in the relatively specific post deep brain stimulation surgery setting (Thobois *et al.*, 2013), while case reports in the literature demonstrate success using dopamine agonists to treat apathy in the post-stroke setting (Kohno *et al.*, 2010; Adam *et al.*, 2013). However, another recent study failed to demonstrate a significant benefit of rotigotine (another dopamine receptor agonist) on apathetic symptoms in Parkinson’s disease (Chung *et al.*, 2016).

Our results, in which apathy and dopamine (depletion) were associated with dissociable changes in the pattern of responding in an effort-based decision-making task, suggest distinct mechanisms may underlie their effect on motivated behaviour. This is concordant with a recent study that assessed the effects of apathy and dopamine on autonomic responses (change in pupillary dilatation) to reward. Apathetic patients showed a reduced autonomic response to reward, and although dopamine increased the magnitude of this response, it did so in both apathetic and non-apathetic groups, such that there was no Drug × Apathy interaction (Muhammed *et al.*, 2016). Similarly, in the current study although dopamine administration was associated with increased acceptance of offers in apathetic (and non-apathetic) patients, it did not change the underlying apathetic pattern of responding, i.e. a failure of low rewards to incentivize behaviour. Together, this suggests a general invigorating effect of dopamine across underlying motivational states, but along a separate mechanistic axis to that underlying apathy.

Apathy and dopamine also showed dissociable effects on response vigour, assessed by the peak motor response at each required effort level. Strong evidence from animal work suggests the dopaminergic system plays a crucial role in appetitive, activational behaviour, i.e. modulating vigour and persistence of action(s) towards a goal (Salamone and Correa, 2012). A recent computational account of dopamine’s effects on choice and response revealed an independent effect on motor activation rate, speeding up the rise of force (yank) to its peak (Le Bouc *et al.*, 2016). In the present study, dopamine depletion reduced peak motor response (for reward) in patients with Parkinson’s disease, independent of reward on offer. Importantly there was no change in the maximal force a subject could produce (MVC) between the two conditions. Interestingly, despite the fact that patients exerted more force ON than OFF medications, their perception of how physically demanding the effort was did not change. The same effect was not observed for apathy; while apathetic patients showed reduced engagement with offers, once an offer was accepted apathy had no effect on the vigour of the squeeze. Additionally, there was no interaction between apathy and the effect of dopamine on response vigour; as with choice, dopamine exerted its effect whether patients were apathetic or not. The findings reported here regarding the effects of dopamine are in line with Le Bouc *et al.* (2016), but use a different analysis approach, while also demonstrating that this effect is general (present across decision space) and crucially independent of the presence of clinical apathy.

Our results demonstrate effort-based decision-making is disrupted in Parkinson’s disease apathy, but in a manner distinct to that associated with dopamine depletion. This does not rule out a role for disruption of dopaminergic systems in the aetiology of apathy, but suggests this alone may not be sufficient to explain the development of the syndrome, nor should it be the sole focus for therapeutic interventions. This conclusion would be consistent with recent reviews of the neurobiology of motivated behaviour and decision-making, which also emphasize the importance of non-dopaminergic neuromodulators (Bailey *et al.*, 2016*a*; Salamone *et al.*, 2016). These include serotonin (Bailey *et al.*, 2016*b*; Maillet *et al.*, 2016), acetylcholine (Fobbs and Mizumori, 2014), noradrenaline (Varazzani *et al.*, 2015) and adenosine (Nunes *et al.*, 2013). Indeed, manipulations of these systems have also shown some promise for the treatment of apathy in other conditions (Rea *et al.*, 2015; Callegari *et al.*, 2016). The heterogeneity in the effects of the dopamine manipulation on choice responses was also a notable feature of this study. An open question is whether those apathetic subjects in whom dopamine depletion had a greater effect on choice, would be more likely to attain a therapeutic benefit from (increased) dopaminergic treatment for their apathy.

Performance on the effort-based decision-making task correlated with the action initiation subscale of the LARS, consistent with previous work examining effort-based decision-making and apathy traits in healthy people (Bonnelle *et al.*, 2016) and reward sensitivity in Parkinson’s disease (Muhammed *et al.*, 2016). Indeed, the questions that comprise this subscale emphasize a person’s daily activities: what they actually do during the day, and the need for prompting in doing daily tasks. These two features are at the core of apathy. Both require a decision to engage, a factor we have explicitly tested in this study. The lack of correlation with other LARS subscales could be interpreted in two ways. Dysfunction of effort-based decision-making mechanisms might be just one of many mechanisms by which apathy can develop. Impairments of executive functions such as planning have also been associated with apathy in Parkinson’s disease (Weintraub *et al.*, 2005), while a recent review emphasizes, in addition to the above factors, the potential contributions of dysphoria (‘emotional distress’) (Pagonabarraga *et al.*, 2015). However, it remains unclear how the different questionnaire components relate to underlying neurobiological systems, the dysfunction of which must drive the development of apathy. An alternative approach to understanding the neurocognitive mechanisms underpinning apathy might be to start from implicated systems, and investigate which of their components contribute to apathy (Holroyd and Umemoto, 2016; Salamone *et al.*, 2016; Le Heron *et al.*, 2017). Here we have demonstrated that apathy is associated with reduced engagement in effortful behaviours for reward, driven by reduced incentivization of low rewarding outcomes. Our findings also suggest that the vigour of motor response is not altered in apathetic patients, once they have decided to engage with an offer. However, there are other aspects of effort-based decision-making this study has not examined—notably persistence towards a goal (Holroyd and Yeung, 2012; Howe *et al.*, 2013; Hamid *et al.*, 2016), learning from the outcomes of actions (Scholl *et al.*, 2015; Holroyd and Umemoto, 2016; Hauser *et al.*, 2017), and tracking background environmental reward information in order to compare the value of current actions with alternatives (Pearson *et al.*, 2014). Future work might profitably focus on whether these neurally dissociable factors of motivated behaviour are also disrupted in apathy, and whether dopamine may play a modulating role. Furthermore, while this study provides behavioural evidence that disrupted decision-making underlies apathy in Parkinson’s disease, future work should focus on linking these behavioural changes to the neural substrates that underlie normal effort-based decision-making. These regions include anterior cingulate cortex and ventral striatum—areas that have already been associated with Parkinson’s disease apathy in separate imaging studies (Remy *et al.*, 2005; Carriere *et al.*, 2014; Baggio *et al.*, 2015; Santangelo *et al.*, 2015).

We found that the presence of apathy was associated with reduced quality of life. The past two decades have seen a surge in recognition of the importance and impact of non-motor features of Parkinson’s disease (Chaudhuri *et al.*, 2006; Aarsland *et al.*, 2009). A recent study demonstrated that, following deep brain stimulation treatment for motor symptoms, those without apathy at baseline experienced a significant improvement in quality of life, while those with apathy did not, despite showing the same degree of improvement in motor symptoms (Martinez-Fernandez *et al.*, 2016). The current study adds further evidence that apathy is an important factor in a patient’s global wellbeing, reinforcing the importance of understanding its mechanisms and developing effective treatments.

In interpreting these results, it is worth considering strengths and potential limitations of this study. The findings suggest that dopamine has a dissociable effect from apathy on effort-based decision-making. However, the dopamine manipulation was within the context of Parkinson’s disease, itself a hypo-dopaminergic condition. This means that even ON dopamine, it is unlikely patients had normal function within dopaminergic pathways. Additionally, although we can conclude that depleting dopamine produces an effect distinct to apathy on effort-based decision-making, we cannot speculate as to whether a supra-therapeutic level of dopamine might in fact have altered the reduced incentivization by low reward outcomes that characterized apathy. Furthermore, as we note earlier, there is evidence implicating degeneration of the mesolimbic dopaminergic system (and particularly structures such as ventral striatum) in the development of Parkinson’s disease apathy (Remy *et al.*, 2005; Thobois *et al.*, 2010; Santangelo *et al.*, 2015), and these changes in themselves may limit the effect of further dopamine administration on the apathetic state. Our study included a number of patients with relatively mild motor symptoms, who consequently were not requiring high doses of dopaminergic medications. This meant the overall magnitude of dopamine manipulation was not as high as some previous studies (Le Bouc *et al.*, 2016). However, the fact we were able to demonstrate robust effects of dopamine on different components of effort-based decision-making suggests this was not a significant issue.

There is also always the possibility of a confounding factor influencing apathetic performance on the task (for example reduced attention). However, the observation that apathetic subjects modulated effort output appropriately, showed specific rather than global alterations in choice behaviour, and demonstrated the same changes in decision time to the value of offers as non-apathetic subjects, makes this unlikely in our opinion. Additionally, our study design controlled for potentially confounding factors on choice such as temporal delay (there was the same delay until the next trial whether an option was accepted or refused) and fatigue (by manipulations listed in the ‘Materials and methods’ section), and baseline levodopa equivalent dose was similar between the groups. Although apathetic Parkinson’s disease patients had a higher level of total depressive symptoms, they did not differ from non-apathetic patients in levels of dysphoria symptoms. Furthermore, in contrast to apathy, dysphoria was not associated with changes in effort-based decision-making. This is consistent with the conceptualization of apathy and depression as dissociable constructs, although constructs that can overlap, particularly within subcomponents of anhedonia—specifically the willingness to work for rewarding outcomes (Pluck and Brown, 2002; Treadway and Zald, 2011; Salamone *et al.*, 2016). Our patient population was drawn from a number of local Parkinson’s disease clinics, and thus is likely to be generally representative of the spectrum of Parkinson’s disease likely to be seen in other general neurology populations.

This study demonstrates specific but distinct effects of apathy and dopamine on effort-based decision-making during both decision and response phases. Apathetic patients showed reduced incentivization by rewarding outcomes, and while dopamine increased their responding, it did not change this underlying response profile. More broadly it provides empirical evidence for a neurobiological substrate to explain the reduced goal-directed behaviour that characterizes apathy—disrupted effort-based decision-making. It also suggests that the motivational deficit seen in Parkinson’s disease is not simply secondary to dopaminergic depletion of mesocorticolimbic pathways, but is better construed as a more complex phenomenon that may encompass both anatomical changes and other neuromodulatory systems.

## Supplementary Material

Supplementary Data

## Abbreviations

LARS: Lille apathy rating scale
UPDRS: Unified Parkinson’s Disease Rating Scale
