# Supplementary material for: Distinct effects of apathy and dopamine on effort-based decision-making in Parkinson’s disease
Source: Brain. 2018 Apr 23;141(5):1455–69. doi: 10.1093/brain/awy110 (PMC5917786; doi:10.1093/brain/awy110)
Supplement: Supplementary Data [file awy110_brain-2017-01847-file007.pdf]

SUPPLEMENTARY MATERIAL | Le Heron *et al*

ANALYSIS OF CHOICE DATA

The primary outcome of this study was choice (accept/reject), and how this varied across the reward/effort decision space between apathetic and non-apathetic patients, ON and OFF their normal dopaminergic medications. We used a hierarchical generalised linear mixed effects model in order to:

a) Apply a nonlinear (logistic) transform to the choice data, which was clearly not normally distributed.
b) Assess all variables of interest (and their potential interactions) within a single model
c) Account for the effects of inter-subject variability (by modelling the random effect of subject)

We used reward, effort, apathy, dopamine and session as our fixed effects variables, subject as a random effect, and choice (accept/reject) as the outcome variable. This meant every valid trial was included in the model. Reward, effort, apathy status (yes/no), dopamine state (ON/OFF) and session (1st or 2nd) variables were z-scored. We implemented the model with the function *fitglme* within MATLAB (MathWorks, USA), using a binomial link function and the Laplace fit method. We compared the full range of possible interactions, from the full model (including all possible interactions between reward, effort, apathy and dopamine) down to just the main effects, selecting the final model using the Akaike Information Criterion (AIC). The full model was ranked 4th out of 2048 possible combinations, however the 3 models above it differed only in the exclusion of the 4-way interaction and one or both of the apathy*dopamine*effort and/or apathy*dopamine*reward terms, none of which were significant. Additionally these models differed from the Full Model in AIC by less than 2 units (the standard criteria for an improved model fit) and the significance or otherwise of all other factors was not changed whatever model we chose. Therefore we elected to report the Full model (**Supplementary table 1 and Supplementary figure 1).** We calculated the significance of the F statistic conservatively, by allowing only 39 degrees of freedom (number of subjects).

Variable	Parameter Estimate	SE	T statistic	F statistic	P value
**Intercept**	2.72	0.28	9.89	97.9	<0.0001
**Reward**	2.40	0.056	43	1849	<0.0001
**Effort**	-1.29	0.049	-26.4	701	<0.0001
**Apathy**	-0.41	0.28	-1.49	2.23	0.14
**Dopamine**	-0.17	0.051	-3.38	11.44	0.0016
**Session**	0.14	0.029	4.79	22.95	<0.0001
**Rew*Eff**	-0.35	0.046	-7.89	62.3	<0.0001
**Rew*Ap**	0.03	0.056	0.48	0.23	0.63
**Rew*DA**	-0.08	0.050	-1.57	2.46	0.12
**Eff*Ap**	0.13	0.050	2.57	6.58	0.014
**Eff*DA**	-0.14	0.047	-2.87	8.24	0.0066
**Ap*DA**	0.03	0.053	0.61	0.37	0.55
**Ap*Rew*Eff**	0.14	0.046	3.02	9.15	0.0044
**DA*Rew*Eff**	-0.14	0.045	-3.13	9.84	0.0032
**Ap*DA*Eff**	0.08	0.048	1.76	3.09	0.087
**Ap*DA*Rew**	0.02	0.051	0.32	0.10	0.75
**Ap*DA*Rew*Eff**	0.05	0.046	1.11	1.23	0.27

Supplementary Table 1. Fixed effects from Full Model

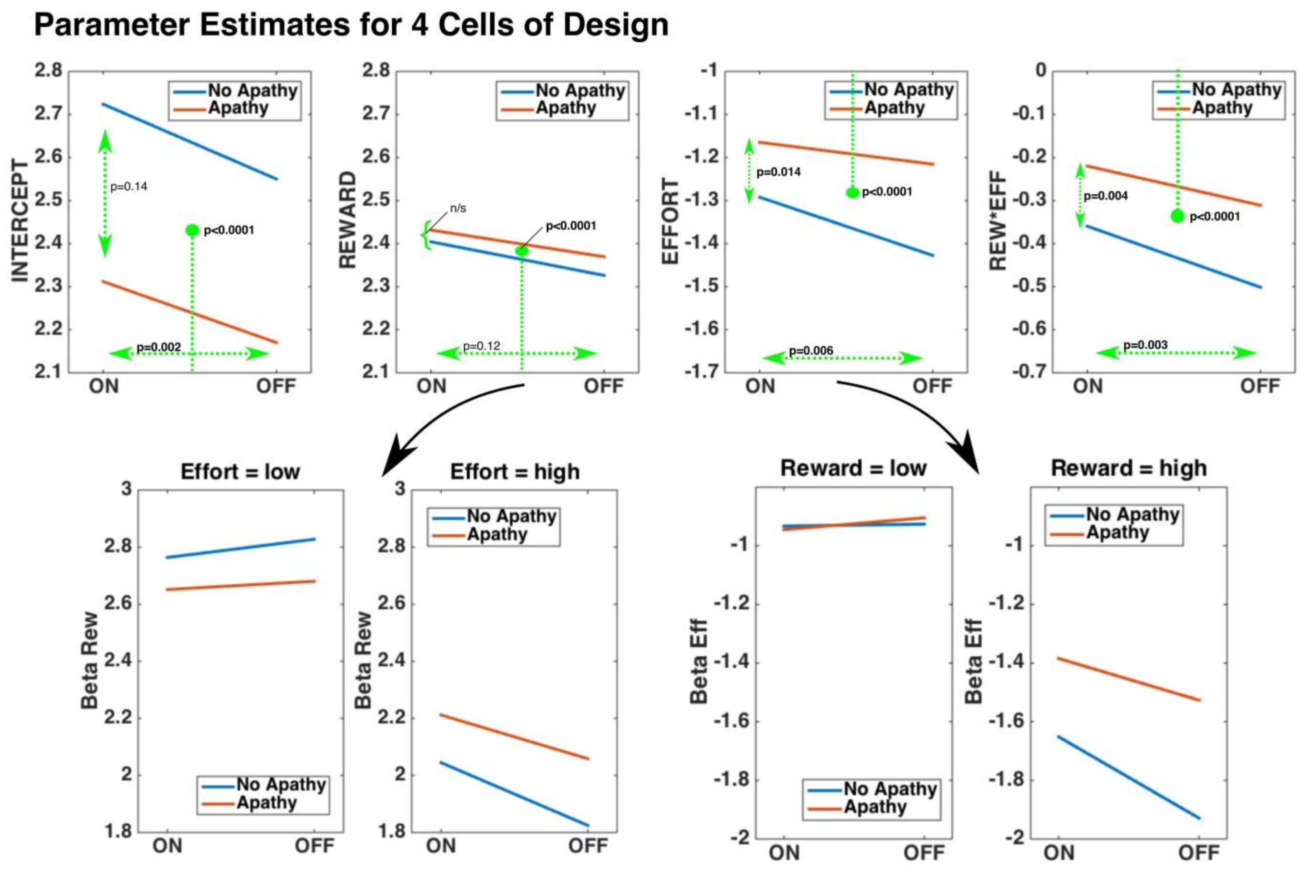

Supplementary figure 1. Parameter estimates for the 4 groups of the design – No Apathy ON, Apathy ON, No Apathy OFF and Apathy OFF.

Top panels: Parameter estimates for Intercept, Reward, Effort and Reward*Effort, for the 4 cells of Design matrix; p values from full model are displayed.

Bottom panels: The two significant 3-way interactions (**apathy*reward*effort** and **dopamine*reward*effort**) are illustrated, by showing how the parameter estimate for reward (left panels) and effort (right panels) varies for high and low effort and reward respectively. **For dopamine**, the *reward* regression slope becomes shallower in the OFF state, at HIGH *effort* levels, whilst *effort* becomes more costly in the OFF state at HIGH *reward* levels. This manifests as a *reduction in choice for High Effort, High Reward offers*. Conversely, **for apathy**, the regression slope for *reward* is shallower at LOW *effort* levels, and steeper at HIGH *effort* levels, whilst *effort* becomes less costly at HIGH *reward* levels. This manifests as a *reduction in choice for predominantly Low Reward (and to some extent Low Effort) offers*.

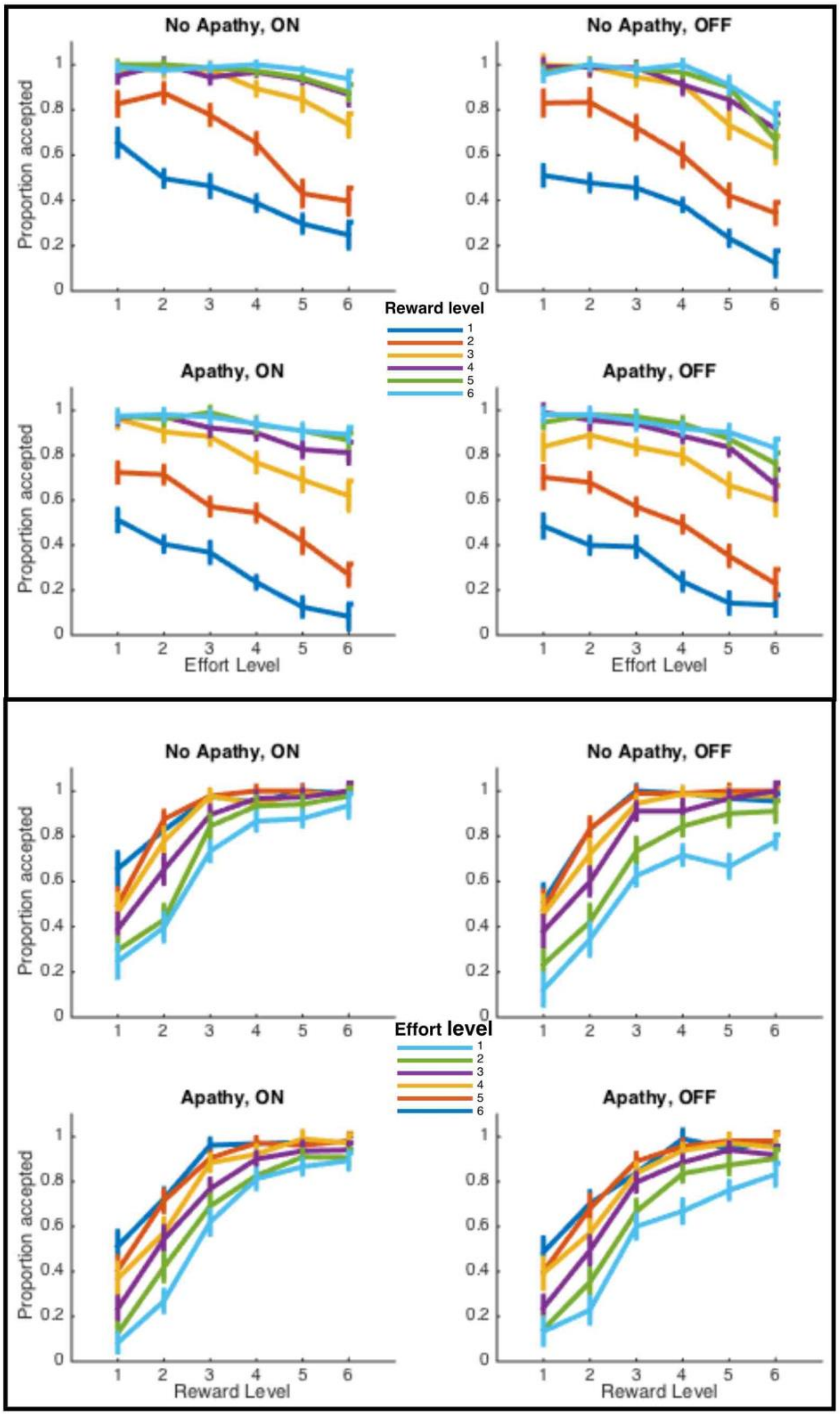

Supplementary figure 2. Proportion of offers accepted for each Reward level, as Effort level increases **(Top Panels),** and proportion of offers accepted, for each Effort level, as Reward level increases **(Bottom panels).**

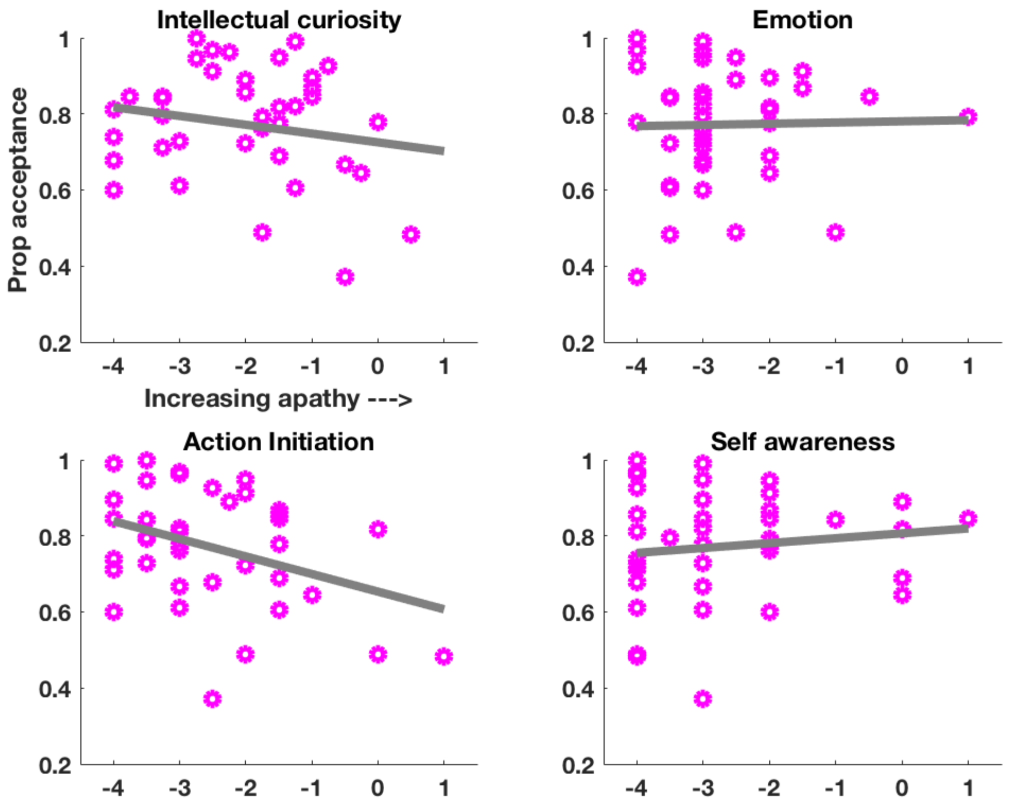

Supplementary figure 3. Relationship between apathy factors and raw proportional acceptance (ON dopamine). Greater impairment on the action initiation subscale was associated with reduced acceptance of offers, whilst no significant effect was seen on the other subscales.

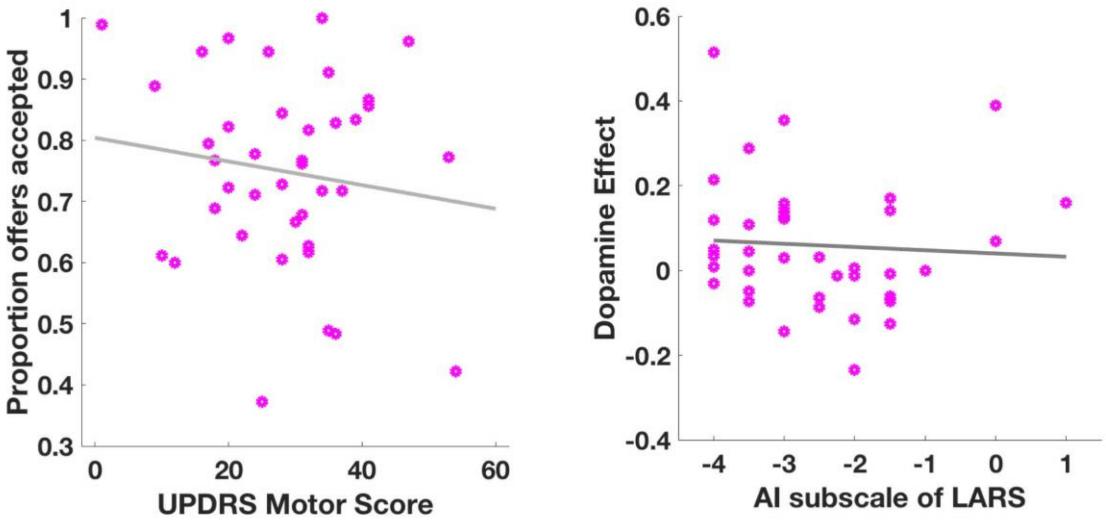

Supplementary figure 4. No significant relationship between offers accepted and baseline UPDRS motor score (ON – R=0.14, N=39, p=0.38, **Left Panel**), or between dopamine effect on choice and the action initiation subscale of the Lille apathy rating scale (R=0.06, N=39, p=0.71, **Right Panel**).

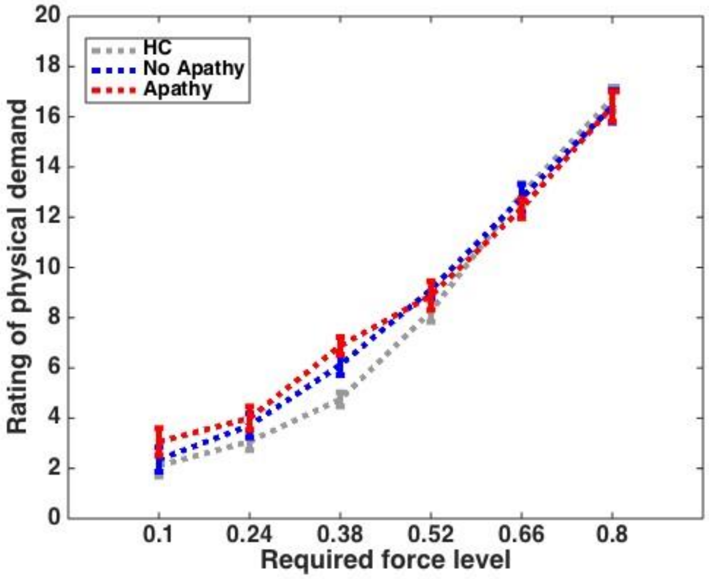

Supplementary figure 5. Subjective rating of how physically demanding each required force level was. No difference between ratings of apathetic and non-apathetic Parkinson's disease patients, or patients and controls.

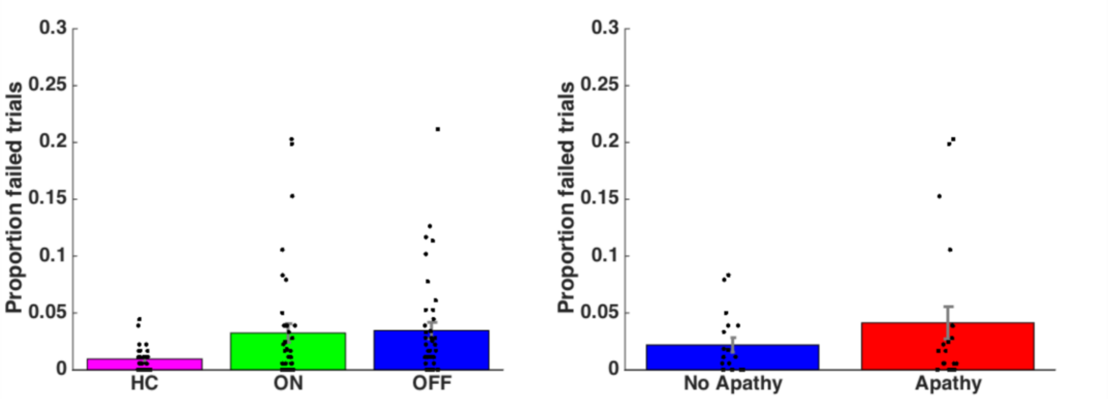

Supplementary figure 6. Proportion of trials subjects failed to achieve force requirement. No differences between *ON & OFF groups* (mean difference (ON-OFF) = -0.002; paired t-test: t_{38} = -0.39, p=0.7); or *no apathy & apathy* groups (mean difference (no apathy – apathy) = -0.0195; unpaired t-test: t_{37} = -1.19, p=0.24).

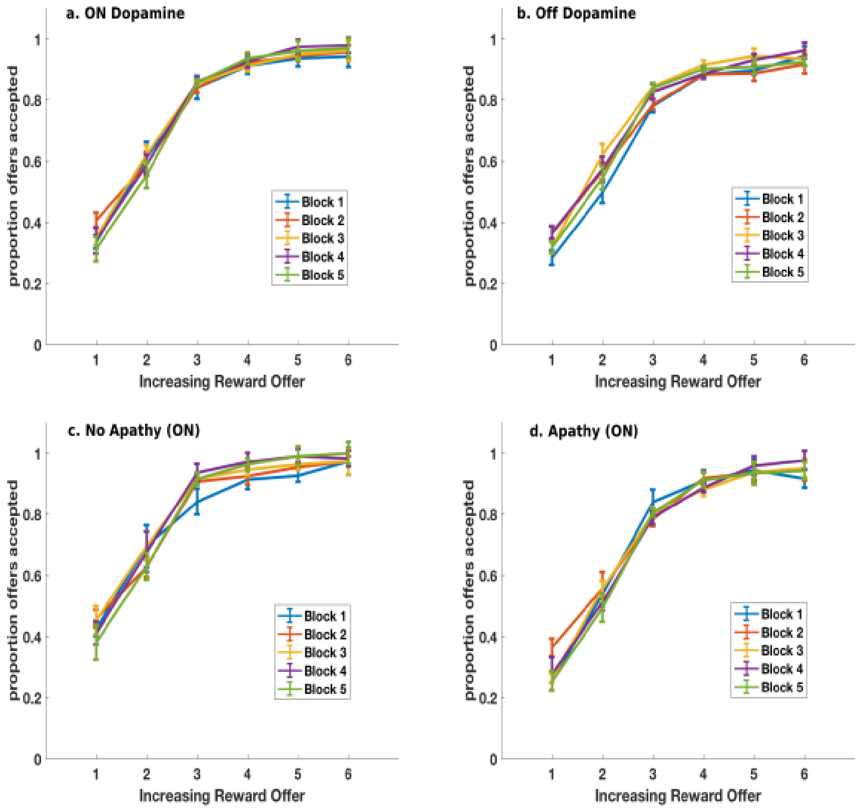

Supplementary figure 7. No evidence of changing acceptance rates across blocks. Proportion of offers accepted as reward increases plotted ON and OFF Dopamine, and for no apathy and apathy patients (in ON state). One-way ANOVA for main effect of block: ON Dopamine $F(4,179) = 0.026$, $p=0.99$; OFF Dopamine $F(4,179) = 0.2$, $p=0.94$; No apathy (ON) $F(4,179) = 0.11$, $p=0.98$; Apathy (ON) $F(4,179) = 0.04$, $p=0.99$).

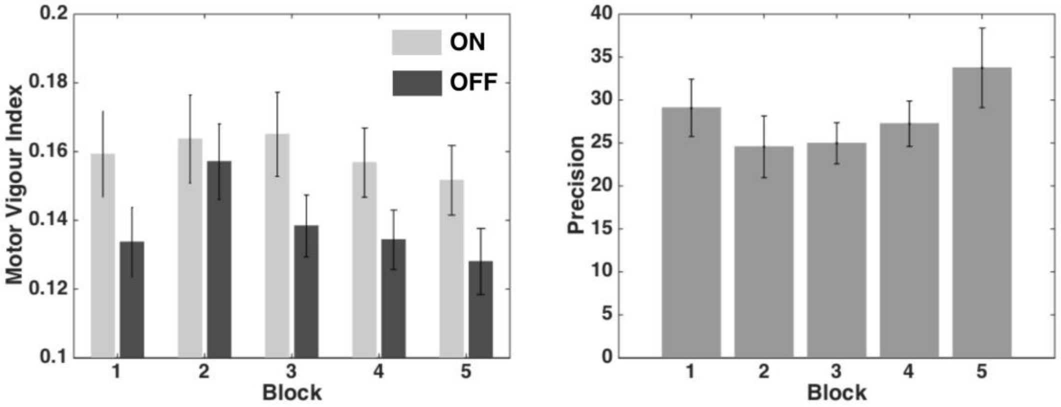

Supplementary figure 8. Effects of Block on Motor Vigour and Precision.

There was a main effect of Block on motor vigour index: F(3.1,118)=3.4, p=0.02, left panel. Polynomial contrasts suggest this effect was not linear (p=0.26) but rather was a quadratic effect (p=0.036), with vigour increasing over the first half of the experiment before then reducing to baseline. There was a main effect of Drug (F1,38)=10.5, p=0.002), but importantly no drug * block interaction (F(3.1, 118)=0.98, p=0.41), meaning the observed reduction in motor vigour OFF dopamine (Main text, Figure 4) was not dependent on the stage of experiment. There was no significant change in precision of motor responses (1/standard deviation) across the experiment, although there is a suggestion of a trend towards improvement in modulating response in the second half of the experiment (F(3,111.2)-1.92, p=0.13, right panel).

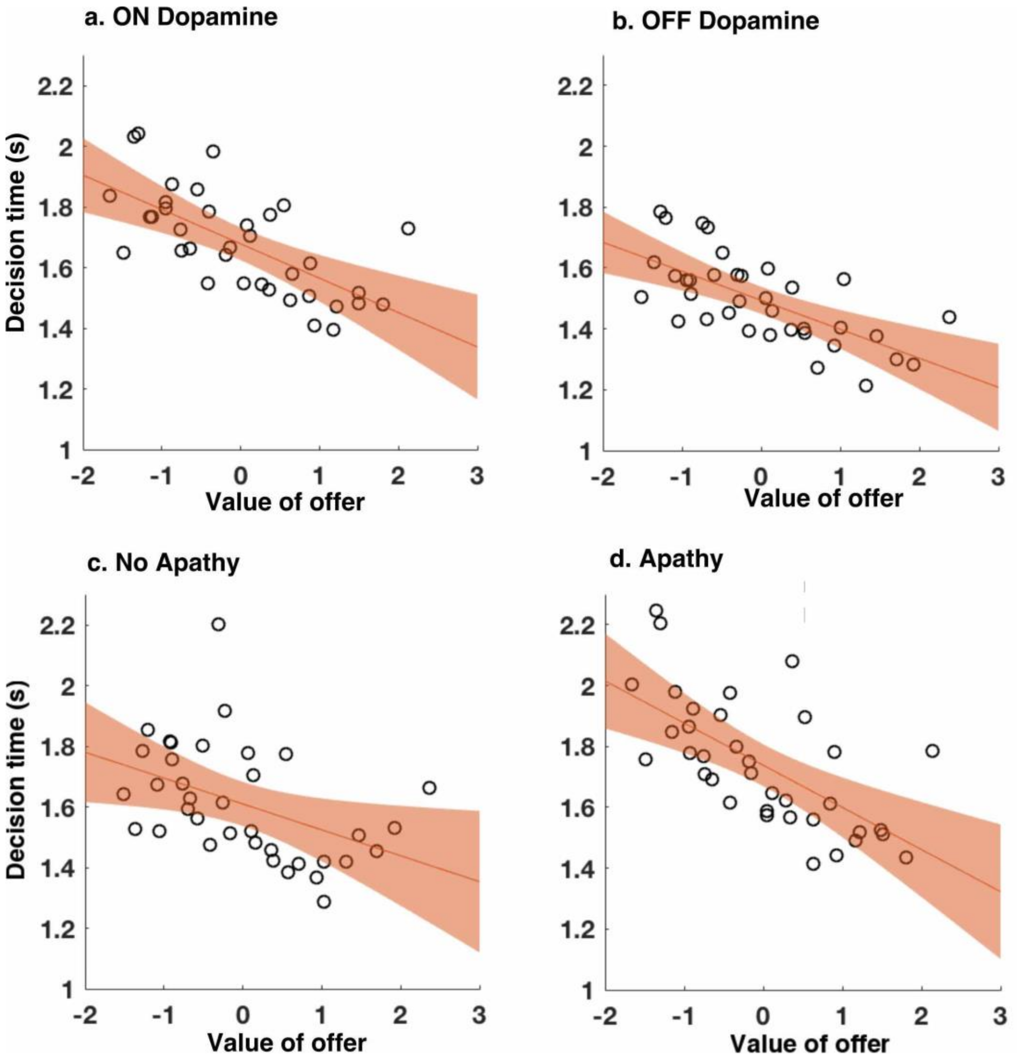

Supplementary figure 9.

Decision time (time to accept or reject an offer) varied across decision space. Value of each reward/effort combination^ (36 in total) is plotted against average decision time ON and OFF dopamine, and with and without apathy. As value of an offer increased , decision time decreased, in both the ON and OFF states (r^2 = 0.45, p < 0.0001; r^2 = 0.46, p< 0.0001, respectively, **a & b**.), and for non apathetic and apathetic patients (r^2 = 0.2, p=0.006; r^2 = 0.43, p<0.0001, respectively, **c & d**.)

^(reward discounted by effort, computed using the parameter estimates of the fixed effects from the generalised linear model used in the primary analysis)

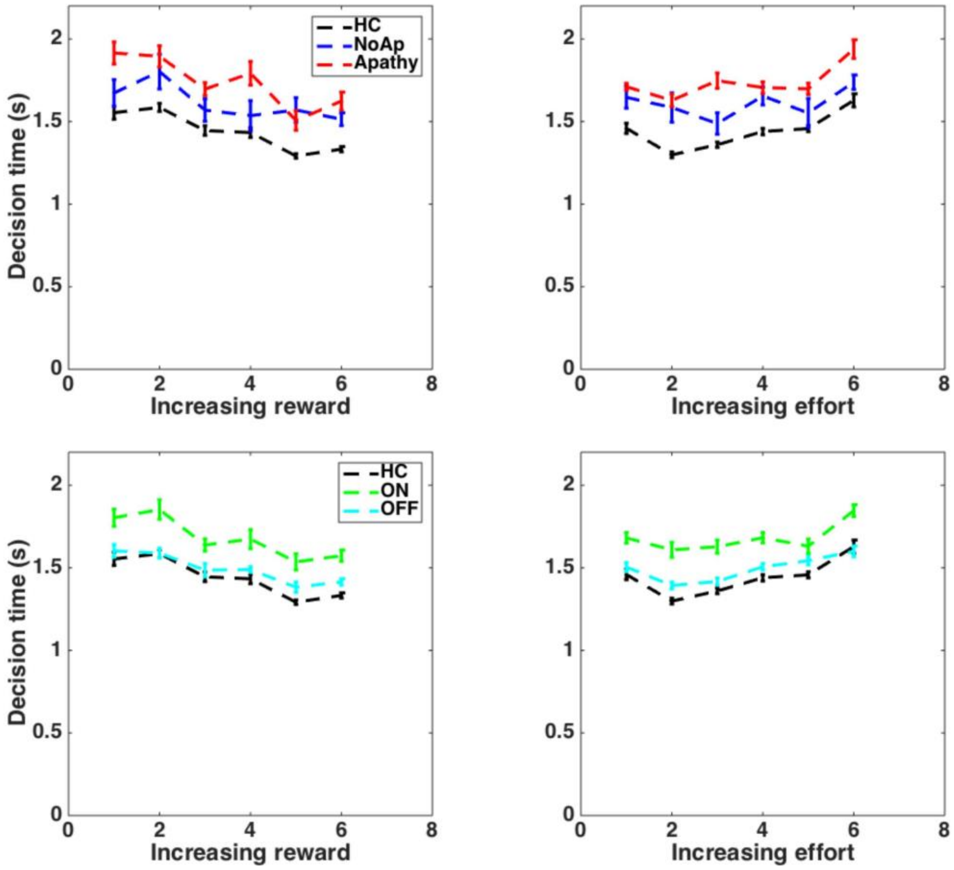

Supplementary figure 10.

There was a significant main effect of both reward (F(3.4,125)=10.3, p<.001 – Left Panels) and effort (F(2.9,109)=7.9, p<0.001 – Right Panels) on decision time. The mean decision time in the apathetic group was 1.67s, compared to 1.49s in the non-apathetic group, however this difference was not statistically significant: F(1,37)=0.97, p=0.35 – Top Panels. Patients OFF their dopaminergic medications trended towards making *faster* decisions (1.67s ON vs 1.48s OFF; F(1,37)=4.04, p=0.052 – Bottom Panels). There was no interaction between apathy and dopamine, nor between these and the other terms.

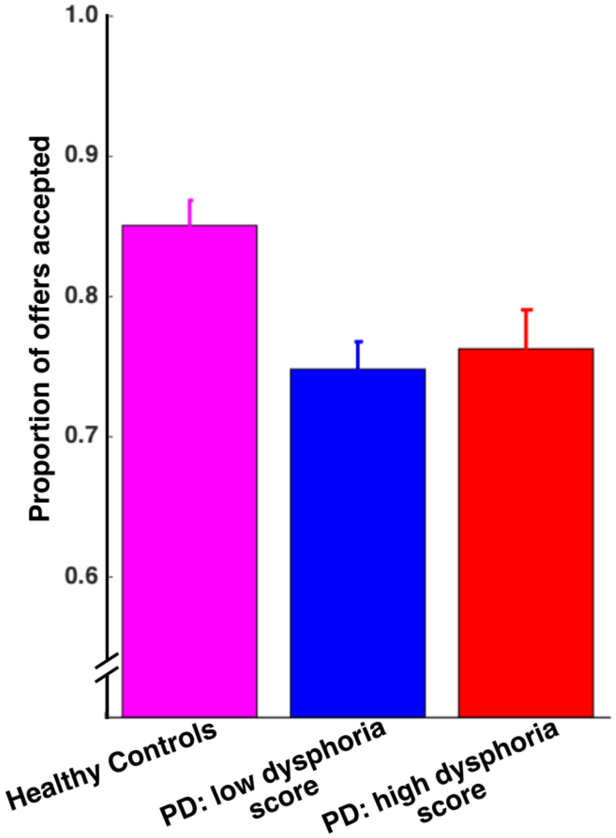

Supplementary figure 11. Higher scores on the dysphoria subscale of the BDI-II were not associated with reduced acceptance of offers ($t(34) = 0.29$, $p = 0.77$). Note groups were divided based on a median split of the data (cut-off subscale score = 4.5). BDI-II scores were not available for 3 patients.
